# Attitudes and experiences of asylum seekers and refugees to the COVID-19 vaccination: a qualitative study

**DOI:** 10.3399/BJGPO.2023.0016

**Published:** 2023-05-17

**Authors:** Anna Clare Talitha Gordon, Caroline Crenstil, Loubaba Mamluk

**Affiliations:** 1 Population Health Sciences, University of Bristol, Bristol, UK; 2 North Bristol NHS Trust, Bristol, UK; 3 The Haven, Sirona Care and Health C.I.C, Bristol, UK; 4 National Institute for Health and Care Research Applied Research Collaboration West, Bristol, UK; 5 University Hospitals Bristol NHS Foundation Trust, Bristol, UK

**Keywords:** refugees, COVID-19, vaccination hesitancy, vaccination refusal, health services accessibility

## Abstract

**Background:**

COVID-19 disproportionately affected asylum seeker and refugee (ASR) populations owing to language and cultural barriers, lower health literacy, polytraumas and mental health needs, and increased exposure. Despite this, there was vaccine hesitancy and low vaccination rates in ASR populations.

**Aim:**

To explore the attitudes to and experiences of the COVID-19 vaccination among ASRs.

**Design & setting:**

Qualitative study of 12 diverse purposively recruited ASRs in Bristol.

**Method:**

Semi-structured interviews were conducted, transcribed verbatim, and analysed thematically to identify emergent themes.

**Results:**

Eight refugees and four asylum seekers were recruited, five of whom were female and seven male, aged between 23 years and 48 years; together representing seven countries. Six were part of a UK Home Office (UKHO) resettlement programme, and six had arrived in the UK by independent means. Analysis showed delayed uptake rather than vaccine refusal owing to the following three main themes: systemic asylum issues (repeated relocation, uncertainty, and dependency on the charity sector); fear (secondary to social isolation, misinformation, and mental illness); and trust (surrounding access to care and community relationships).

**Conclusion:**

Fear, trauma, and isolation propagated by systemic issues are primary factors impacting healthcare decision making, and standard approaches to increasing vaccination uptake must be reconsidered in light of these issues. General practice must appreciate and invest in providing security in healthcare access for ASR populations. Barriers to practice registration must be overcome to enable ASRs to access care both around vaccination and afterwards. Communication must be clear and accessible to aid individuals in making informed decisions, balancing the benefits and potential risks of vaccinations.

## How this fits in

Previous literature anticipated vaccination refusal in ASR populations owing to misinformation and poor practical access to vaccination. This study qualitatively describes hesitancy rather than refusal, contributed to by multiple factors. It was discovered that although hesitancy was widespread, it was related to systemic asylum issues such as housing instability and asylum case delays, fear contributed to by previous traumas both in the UK and overseas, and trust (or lack thereof) around access to care at community-wide rather than individual levels.

## Introduction

In 2022, there were >350 000 ASRs in the UK.^1^ One-third were awaiting asylum decisions from the UKHO. These individuals are asylum seekers (AS) who arrived in the UK by independent means; outstaying an existing visa or entering by undocumented routes.^1–3^ The remaining two-thirds have received positive asylum decisions and are refugees. Within this, some are resettled refugees (RR), part of a UKHO scheme that selects and transfers vulnerable families from conflict zones to the UK. Assistance with housing, income, employment, health care, and education is provided by local councils, communities, and charitable organisations.^2–4^

The ASR population is diverse, dynamic, and non-homogenous, with the exception of the asylum system they have in common. There are acute differences between RRs and those who are 'independent' ASRs (IASRs). This article explores the impact of being an ASR on attitudes to and experiences of the COVID-19 vaccine, comparing the differences between RRs and IASRs.

The COVID-19 pandemic disproportionately affects ASRs owing to increased disease prevalence and severity.^1^ This is exacerbated by frequent relocations, overcrowding, and homelessness, limiting their ability to lock down effectively.^5^ Despite this, vaccination hesitancy is common, and vaccination rates are below the national average.^6^ There is minimal published data exploring ASRs’ attitudes to or uptake of the vaccination, although literature has forecast contributing factors based on barriers to primary care, and those impacting other minority populations.^7^

Barriers to primary care for ethnic minority populations include miscommunication caused by language barriers because of inadequate provision of interpreter services, lower health literacy, cultural differences around care-seeking and expectations, and traumatic previous experiences of health care.^8^ In ASRs, this is compounded by poorly understood complex mental health and social needs, a lack of knowledge among ASRs and healthcare staff of legal entitlement to health care, and fears that seeking health care may compromise an asylum case.^9,10^ In the UK, this is complicated by policies charging refused AS for health care.^11–13^ The pandemic and lockdown curtailed already limited charitable, health, and social services, isolating ASRs from face-to-face assistance with medical, legal, housing, financial, and social issues, and adding significant delays to asylum procedures.^11^ The COVID-19 vaccination was introduced on this background of heightened perceived and real barriers to health care.

The UK vaccination roll-out demanded and created hierarchy-flattening strategies and collaborations, and increased community partnership to achieve high uptake at speed.^7,11,14–19^ Interventions were targeted to ASRs based on predictions that vaccine hesitancy would be associated with populations with lower incomes and literacy rates, Muslim religion, or minority ethnic groups.^3,6,16^ The interventions primarily aimed to combat vaccine misinformation circulating on social media platforms (WhatsApp and Facebook), utilised by minority groups and particularly impacting those with low literacy.^7,11,20–24^

Although interventions were based on evidence and local knowledge, greater understanding of continuing structural and individual barriers to vaccination and accessing health care for ASRs is required. This study sought to understand the attitudes and experiences of the COVID-19 vaccination in the ASR population.

## Method

### Qualitative branch

#### Study design and research team

Semi-structured interviews were conducted with 12 ASRs in Bristol attending one of three non-governmental organisations (NGOs) providing education, community support, or food. In September 2021, the lead author volunteered at all three NGOs for 2 weeks and met with potential participants and staff. Recruitment then began face to face using quota and snowball sampling in collaboration with NGO staff to ensure only those appropriate to interview in terms of mental health and age (>18 years) were approached. Interested individuals were given information sheets in their first language and opportunities to ask questions 7 days before their interview. Written consent was documented immediately before interviews. No participants dropped out following recruitment. A highly diverse sample in ethnic group, age, sex, and English-speaking ability was recruited.^23^

#### Data collection

Topic guides (written in English) were formulated iteratively, informed by a literature review and the previous experience of the lead and third author, and later by previous interviews. Interviews were face to face, lasting approximately 30 minutes, and conducted by the lead author (who is trained in interview technique). The interviewer was introduced as a *'researcher from the university, collaborating with the city council to understand their experiences'*. Interviews were in English or with an interpreter as chosen by the participant. Six Arabic-speaking participants requested an interpreter, for whom a female native Arabic speaker interpreted responses verbatim into English, which was recorded, checked by the third author (a native Arabic speaker) for accuracy, and transcribed in English. The interpreter was formally trained, worked regularly with the NGOs and the city council, and was familiar to participants. Interviews were conducted in private rooms in NGO buildings or at participants’ homes with only one researcher (the lead author), participant, and where requested, an interpreter, present.

Interviews began with closed demographic questions followed by broad questions establishing knowledge of the disease and vaccination, access to care, and lived experience, before more specifically exploring barriers and facilitators to care. Finally, it was asked whether participants had or had not accepted the vaccination and why. Data collection continued to saturation on these key topics.

Interviews were digitally recorded using an encrypted device, transcribed verbatim, and screened for accuracy before being made anonymous by the lead author. Field notes to provide context to interviews and a reflexive diary for self-awareness were maintained throughout data collection and analysis. This enabled analysis of setting, interactions, and non-verbal cues during and around interviews.

#### Data analysis

Data were organised using NVivo (version 12) and reflexive thematic analysis undertaken with an inductive, semantic, and critical approach.^25–27^ Codes were derived from the data by the lead author and independently verified by the third author, with consensus reached regarding themes in regular analysis meetings. Member-checking of transcripts was not pragmatic owing to low-written English literacy for many participants and privacy, as contact details were not collected.

Funding constraints meant participants were unable to be offered financial incentives.

## Results

The 12 ASRs, seven female and five males, aged between 23 years and 48 years, and representing seven nationalities, are described in Table 1. Eight were refugees and four AS. Six were RR and six IASR; one outstaying a student visa, and five entering the country via undocumented routes (Figure 2)*.* Despite only two participants reporting receiving information regarding the vaccination in their own language, 10 had received at least one dose of the vaccination.

**Table 1.** Demographics of participants

Participant number	Sex	Age, years	Nationality	Years in UK	Asylum status	Entry to UK
1	F	23	Eritrea	5	Asylum seeker	Undocumented route
2	M	40	Bangladesh	26	Asylum seeker	Undocumented route
3	F	30	Iran	1	Asylum seeker	Undocumented route
4	M	31	Iran	1	Asylum seeker	Undocumented route
5	F	30	Taiwan	7	Refugee	Outstayed visa
6	F	30	Somalia	7	Refugee	Undocumented route
7	M	48	Iraq	0	Refugee	Resettled
8	F	40	Iraq	0	Refugee	Resettled
9	M	38	Iraq	4	Refugee	Resettled
10	F	30	Iraq	4	Refugee	Resettled
11	F	30	Syria	5	Refugee	Resettled
12	M	40	Syria	5	Refugee	Resettled

F = female. M = male.

Eight ASRs were housed in private local council accommodation, two in temporary accommodation, and two were informally ‘sofa surfing’. Three described being moved between cities by the UKHO, and seven had lived transiently in other countries before entering the UK (Figure 2).

**Figure 1.**
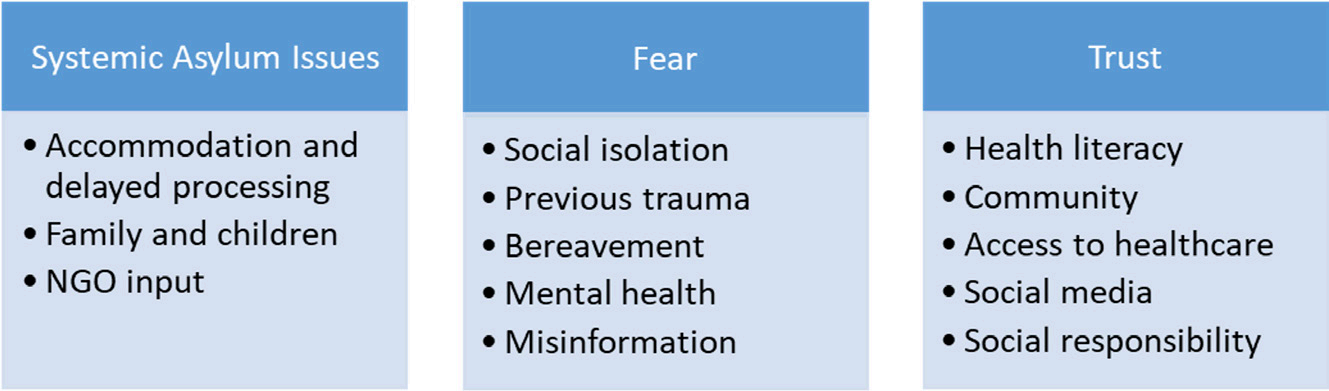
Themes and subthemes. NGO = non-governmental organisation.

The findings were consistant with three themes and subthemes (Figure 1)*.* Throughout interviews, although questions focused on vaccination, participants responded by discussing their experiences with healthcare services as a result of the asylum system. The themes therefore reflected the breadth of interview responses; however, a summary of key barriers and factors increasing uptake induced from this can be seen in Table 2.

**Table 2.** Factors affecting uptake of vaccination

Factors increasing uptake	Barriers to vaccination
Sense of social responsibility in community	Systemic asylum system issues
Good, accessible information	Misinformation
Desperation to escape lockdown	Poor mental health
Trusting relationship with GP	Trauma and bereavement overseas
NGOs assisting access to health care	Perceived poor access to care
Sense of community	Social isolation
Fear of COVID-19 greater than fear of vaccination	Fear of vaccination greater than fear of COVID-19

NGOs = non-governmental organisations.

### Systemic asylum issues

#### Accommodation and delayed processing

**Figure 2.**
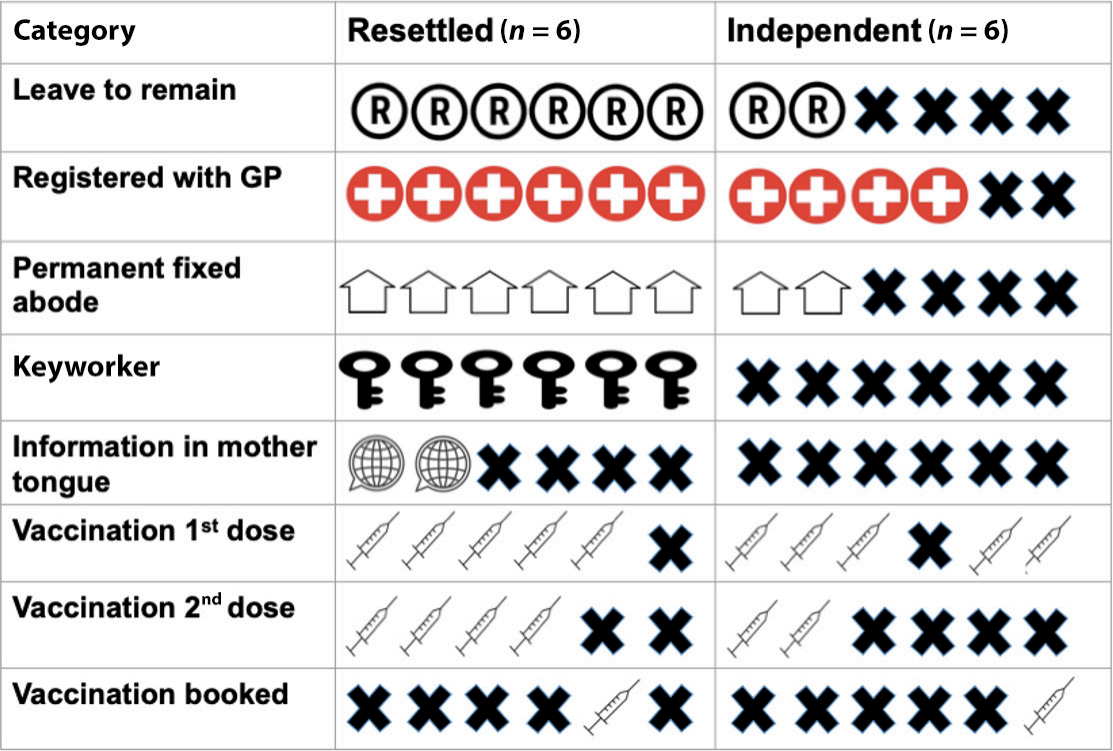
Resettled and independent refugees key indicators.

There was a stark divide between the six RRs and six IASRs.^21^ Compared with all six RR, none of the IASRs were keyworkers, one in three were living in accommodation that was ‘permanent’, and two in three were registered with a GP (Figure 2). One IASR described their 25 years in England as follows:

*'... from 1995 by myself or with some friends, no family members or anything here, which is a big problem. Sometimes with my friends here living here or there, sometimes house-sitting … sometimes I’m a little bit down, it’s very difficult for me.'* (Participant [P]2, AS)

The UKHO failed to provide secure and acceptable accommodation for some ASRs. Examples included long periods in temporary hotels, forced relocation between cities, inadequate housing, and voluntary homelessness to remain geographically near support networks (family and/or friends) rather than having guaranteed shelter at locations further afield. During the pandemic, slower processing of asylum claims lengthened stays in temporary accommodation and resulted in increased numbers of ASRs housed there. One participant said:

*'... there are a lot of people now in the hotels. My friend is living around more than 14 months in a hotel … with an 11-year-old son … waiting in a little room … After these fourteen months he’s so nervous and he has anxiety and both of them nowadays are so depressed.'* (P3, AS)

An AS participant reported that:

*'Every time when you called the UKHO, they said in this Coronavirus period. You must wait. We know you have a problem, but you must tolerate it.'* (P4, AS).

#### Family and children

Seven participants described additional strain of supporting children with a lack of practical and psychosocial support. This was heightened for families resettled because of the illness of a child. One mother described their feelings as follows:

*'... it will destroy us … all the time we’re at home … we feel scared because a lot of people they said to us, you have a responsibility, your child is sick, and you want him to get sick?'* (P9, RR)

#### NGO input

IASRs described the positive impact of NGOs, and RRs described how they felt supported by their keyworkers in their resettlement programme; for example, one RR commented:

*'...* [Keyworkers] *made it more easy for us. They was with us step by step. If we need any information, they explain it, so that’s what made it more easy for us.'* (P7, RR)

Keyworkers and NGOs helped with accessing, translating, and interpreting information, transportation, and registering with GPs. IASRs were often dependent on NGOs for financial, social, and emotional support, alongside signposting to housing, healthcare, and legal services.

### Fear

#### Previous trauma and bereavement

Participants described fear provoked by traumas before claiming asylum. Nine participants described losing family members and friends to COVID-19 both in the UK and overseas. One participant described awareness that:

*'... a lot of community members they lost their life ... a big number.'* (P6, independent refugee [IR]).

In addition, participants described traumas when seeking health care in other crises or war zones; for example, one participant said:

*'... you know like in Iraq if someone gets sick and he goes to hospital, he will not come back. He will die.'* (P10, RR)

These traumas and resultant fears, alongside the national and international news and social media, were overwhelming, as shown by the following comment:

*'... can’t stand anymore to just stay with these things. I couldn’t travel outside, couldn’t go anywhere, I lost some of the relatives in Iraq they died of the disease and here friends they died so all this affected me.'* (P9, RR)

#### Mental health

Traumas and fears contributed to diagnosed mental illness detailed by nine participants. Four of the nine participants disclosed that they were taking medication or had sought specialist help, despite multiple participants commenting that mental health was not widely discussed in their communities. For example, one IR stated:

*'... we don't really want to tell people, "*[I] *have a mental health issue".'* (P5, IR)

#### Social isolation

In some families, fear manifested as remaining in self-imposed lockdown for up to 18 months longer than national policy required. One participant said:

*'We just stayed in our tiny flat. Nothing more than. Three months we didn't go out.'* (P9, IR)

UKHO accommodation that was far from support networks also contributed to isolation. People often stayed for prolonged periods, and had the dual threat of the pandemic and prolonged uncertainty regarding their future, owing to delayed asylum-case processing times.

#### Misinformation

Social isolation owing to fear, lockdowns, and isolating accommodation, alongside reduced health literacy because of language and cultural barriers, left participants vulnerable to misinformation. All participants accessed information online through national news outlets, social media, or by word-of-mouth (including WhatsApp groups). Participants reported reading that COVID-19 vaccinations were *'poison … from China'* (P9, RR), *'make diseases'* (P8, RR), and *'kill you … or* [that] *you can’t have a baby in the future'* (P4, AS).

Despite this, 11 participants were able to identify misinformation that they had seen as untrue, stating that it was *'fake news'* (P4, AS) from *'people who are less educated … just gossip, they don’t know what they are talking about'* (P9, RR).

Participants cited BBC and British formal media outlets as reputable information, over outlets from their countries of origin, or community social media groups, and 10 had already had their first dose of vaccination. One participant refused vaccination owing to online misinformation. They also denied the existence or severity of the pandemic, saying that they had *'heard about a lot of people died and big numbers … they’re lying. It’s not true'* (P12, RR).

As such, they did believe that vaccination was necessary, and vaccines were made by *‘the mafia, they just want to get money from the people'* (P12, RR). This individual had complex social and medical needs rendering them housebound. Their wife was not housebound, and had received the first dose of the vaccine. All participants reported that most people had received their first vaccination. For example, one RR commented:

*'... the majority of* [their community] *has got at least one jab.'* (P9, RR)

Misinformation was unanimously a significant *'reason* [participants] *felt scared'* (P8, RR) of vaccination, particularly regarding side effects or medical vaccination complications. This was the predominant reason for vaccine hesitancy, not barriers to practical access. One participant said:

*'I was worried before, I was hesitant before I take the vaccine so I was waiting if I can see somebody who had the vaccine and is still alive*.*'* (P6, IR)

### Trust

#### Health literacy

Three individuals who worked in health care or had attended university actively researched and identified misinformation based on their health literacy; for example, an AS said:

*'... when I first I heard* [about the vaccine] *I had no idea about that, but I did some research and ... thought yeah, that’s necessary, we should do that because we can protect ourselves and others*.*'* (P4, AS)

Eight other participants based these decisions on the viewpoints and actions of trusted individuals felt to be educated and informed. One participant reported finding trust in the vaccination following decisions of *'good friends … they study here PhD, so they took the vaccine — so when I saw that I was like OK'* (P10, RR). Participants also unanimously trusted medical practitioners; for example, one stated:

*'... we heard different opinions from different places, but we are not better than the doctors. All the doctors in general they understand better than the rest of us. And a lot of people they took it and they are OK. Then that’s enough.'* (P9, RR)

All participants described relative ease in overcoming language barriers to information online; for example, one AS commented:

*'…* [it was] *easy, so easy, because even if you cannot understand English, you can use Google translator. So yeah, not perfect, but at least it’s an idea of what’s going on*.*'* (P4, AS)

Specific terminology was often not understood however, and participants varied in the depth of research they attempted to conduct.

#### Community

Regardless of approach to decision making, 10 participants referred to vaccination uptake decisions in terms of *'we'* in reference to their community, and not as an individual. Eleven participants discussed being hesitant rather than refusing to be vaccinated, as this enabled them to *‘wait for somebody to see, and see if they can take and not have side effects or whatever’* (P6, RR). Individuals described sharing vaccination status with communities, with one participant saying:

*‘I ask you, you’ve got the jab, you said yes, of course I've got twice, so I ask another one, “Have you got the jab?” “Yeah, yes, I’ve had it once," so it kind of encourages* [me that] *everyone has got, why I didn't?'* (P4, AS)

#### Social media

Participants who felt isolated communicated through social media with a virtual community of ASRs in the UK and overseas. Participants described large WhatsApp groups spanning multiple countries, which enabled them to *'*c*ompare what’s different, why they have different opinions'* (P5, IR).

One RR described information posted (in Arabic) by a local councillor on a large community WhatsApp group as *'very clear about how many people they could host at home, when they meet all this stuff, how to look after yourself to not get sick from Corona … '* (P10, RR).

This local Council WhatsApp group was also a place they were encouraged to use *'if you need anything … any help and support … go there and ask. You will find a lot of people'* (P10, RR).

This direct communication with the Council enabled the sharing of accurate, relevant information in an accessible format and language.

#### Social responsibility

The heightened sense of community extended to the widely cited rhetoric *'stay home,* [to] *save lives'* (P6, IR).

Participants expressed pride in efforts to protect their communities; for example, one IR said:

*'... we want the society become better be better. Back to normal. So all of us, finally, we decided to have a vaccination*.*'* (P5, IR)

#### Access to health care

Access to care was a crucial determinant of vaccination. Although the motives or ability of healthcare staff were unquestioned, participants felt that they *'don't understand the system, so we not don’t trust the NHS'* (P5, IR).

Participants felt rebuffed by the 111 service, emergency departments, and GP reception staff; for example, an IR stated:

*'*[GP] *receptionist*[s] *always want to hang up your phone … so that’s why some of us we are very afraid to get a vaccination because the if we can't sort out the side effect, maybe we just stay home and extend the lockdown time.'* (P5, IR)

Despite this, 60% of participants who were alread registered with a GP cited their first choice source of vaccination information as their practice, while unregistered participants cited a receptionist:

*‘"It is a pandemic we couldn't register you" ... this time we need the doctor especially.'* (P3, AS)

Five RRs expressed confidence in the UK government; for example, one commented:

*'I feel that the government cares about the people ... If something happened to my family, we will get support. And to prove that, the vaccine in Jordan, we refused to take it until we arrived here. Then I said I'm going to take it.'* (P7, RR)

In contrast, no IASRs expressed this confidence, although they trusted individual practitioners.

Finally, participants struggled to communicate remotely with healthcare services, saying:

*'... if I got anything in a post, it means I cannot reply. I find it hard to write and read this and again to post it.'* (P8, RR)

*'... didn't know anything about how to use the email … but WhatsApp I will just do it easily.'* (P7, RR)

## Discussion

### Summary

In-depth interviews were conducted to explore experiences and attitudes to vaccination. Responses, on analysis, illustrated how decisions around vaccination uptake are a holistic reflection of perceived and real access to health care, and the impact of an asylum system that often fails to provide safety or security to this vulnerable and isolated population. Factors that may increase uptake of the vaccination must also be holistic. There needs to be an awareness of the true impact of the asylum system on individuals within it among practitioners and commissioners; a focus on improving access to primary care; and efforts made to enable individual ASRs to connect with members of their natural communities by ethnic group, rather than simply targetting ASRs with specific interventions focused only on vaccination.

**Figure 3.**
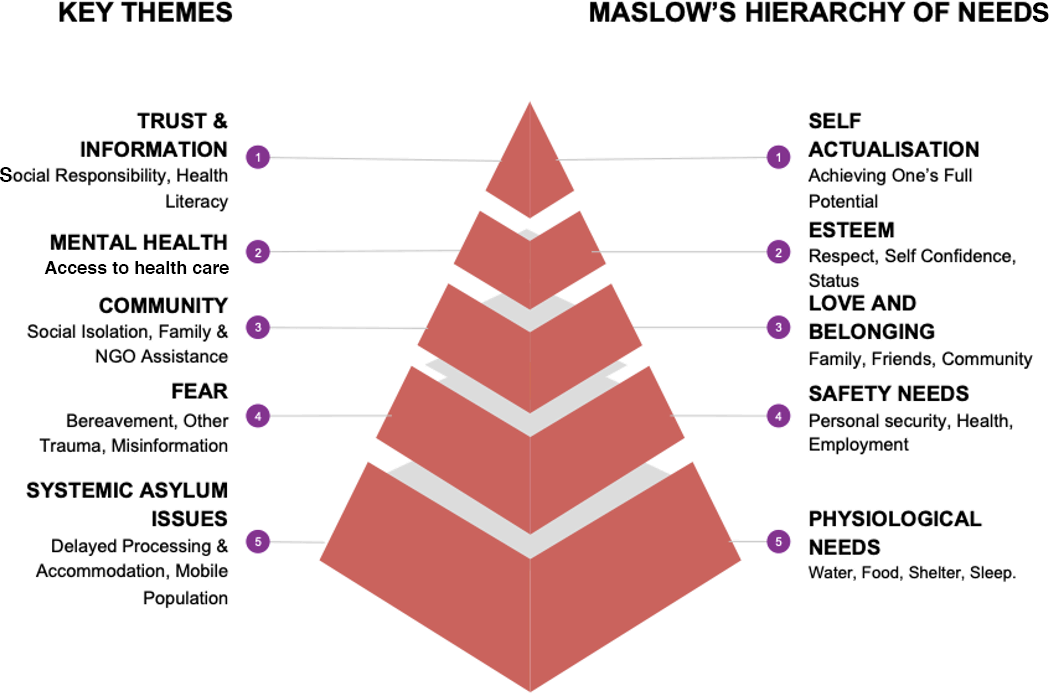
Maslow’s Hierarchy of Needs^28^ and interview thematic analysis. NGO = non-governmental organisation.

Findings were disseminated with local policymakers, commissioners, and practitioners through verbal presentations, meetings, and a lay report. During this process it was discovered that framing the key themes and subthemes alongside Maslow’s Hierarchy of Needs enabled holistic responses to be best presented in a format well understood by practitioners (Figure 3).^28^ The premise of this is that each ‘need’ can only be addressed once the one below it has been fulfilled.

At base, ‘physiological needs’ level 5, all participants could prioritise health care or vaccination while their accommodation and physiological needs remained under threat owing to systemic asylum issues. At the ‘safety needs’ level 4, participants were unwilling to accept a vaccination that could risk their health, the side effects for which they could not access health care to address. Access to a keyworker for RRs, or a good relationship with their GP for IASRs, was the largest factor in participants feeling that this need was met. At the ‘love and belonging’ level 3, participants described the fear bred by isolation, amplified by the pressures of protecting children, and discussed their vaccination status in terms of their whole community. Until there was group consensus that vaccination was safe and participants felt supported in taking risk, they were unlikely to accept vaccination (including those with university education, employed in health care, or healthcare workers’ support). Mental health issues expressed by nine participants added barriers at the ‘esteem’ level 2, as participants stated that they could not process information under pressure or have the confidence to make decisions involving risk.

In summary, participants delayed vaccination unless they felt secure in their accommodation; they felt secure in their access to health care; they felt their decision was supported by their community; and they felt secure in their mental health. Eleven participants were able to identify and cite misinformation that they found frightening but this was less significant in causing vaccination hesitancy than factors described in Figure 3.

### Strengths and limitations

To the authors’ knowledge, this study is the first to explore in-depth the experiences of this population group surrounding the COVID-19 vaccination. The small study population, although non-homogenous demographically, has the impact of the asylum system in common. Key themes therefore have ‘transferability’ to other resettled and IASR populations in the UK moving through the asylum system. The interview setting created a supportive and safe environment, enabling in-depth, personal explorations and interviews to continue until there were almost no new viewpoints expressed and data saturation was reached on content from the topic guide.

Recruitment was, however, limited to individuals engaging with NGOs and, therefore, there may have been more collaboration with healthcare services, which may have impacted the experiences and uptake rate reported. In addition, the study focused on holistic approaches with a small, non-homogenous sample. Further exploration of specific factors impacting uptake and suggested interventions in each ethnic group is necessary. Finally, member-checking was impossible to triangulate findings with participants but data were self-consciously analysed to ensure credibility and dependability, with independent verification of themes and interpretative challenge in analysis meetings.^23^

### Comparison with existing literature

High prevalence of vaccine hesitancy, defined as *'delay in acceptance or refusal of vaccination despite availability of vaccination'* was forecasted in qualitative studies of ASR populations before vaccination roll-out.^22^ Hesitancy has been described in terms of *'confidence'*, *'convenience'*, and *'complacency'*.^22^ Confidence was predicted to be eroded by inaccessibility of information owing to language barriers, misinformation, and negative healthcare experiences.^6,21,22^ Perceived or real practical barriers (including cost), uncertainty around vaccination entitlement, and disrupted NGO services were predicted to decrease convenience.^6,7^ Finally, complacency was predicted to be influenced by slow vaccine uptake among healthcare staff from ethnic minority groups.^2,3,29^

Although findings support predicted hesitancy, barriers surrounding confidence and convenience reflected the far-reaching impact of the asylum system and underlying traumas of ASRs. The present study found no evidence of complacency. The authors suggest these labels seem inappropriate when applied to a fearful and clinically vulnerable population with limited access to health care. Vaccine hesitancy was not because of a lack of information, misinformation, or practical access, but these factors were compounded by social isolation, trauma, and an asylum system, which often fails to provide adequate shelter or safety.

Population health is dependent on equitable access to health care. The COVID-19 pandemic highlighted systemic health inequalities affecting minoritised and marginalised communities. Infrastructures and systems must be rooted in the hierarchy of needs, and priorities of individuals and those targeting ASRs must be designed specifically in light of the asylum system within which they dwell.

### Implications for practice and research

The findings have three implications for clinical practice. First, general practice and policymakers must provide perceived and true equitable access to care for vulnerable populations. This enables them to take the perceived ‘risk’ of accepting vaccinations with confidence they can access care for side effects. Barriers to practice registration must be eradicated.

Second, fear, trauma, and isolation determine ASR healthcare decision making. Increased communication, including resolving issues with remote communication, can assist people to make informed decisions. Utilising WhatsApp or text messaging rather than postal communication enables online translation, if communicating in an individual's first language is not possible.

Finally, research is needed with homogenous groups of ASRs exploring specific experiences of ethnic community groups to which these individuals identify as belonging.
